# Molecular and Biochemical Pathways of Catalpol in Alleviating Diabetes Mellitus and Its Complications

**DOI:** 10.3390/biom11020323

**Published:** 2021-02-20

**Authors:** Subrat Kumar Bhattamisra, Hui Min Koh, Shin Yean Lim, Hira Choudhury, Manisha Pandey

**Affiliations:** 1 Department of Life Sciences, School of Pharmacy, International Medical University, Bukit Jalil, Kuala Lumpur 57000, Malaysia; 2 School of Pharmacy, International Medical University, Bukit Jalil, Kuala Lumpur 57000, Malaysia; koh.huimin@student.imu.edu.my (H.M.K.); lim.shinyean@student.imu.edu.my (S.Y.L.); 3 Department of Pharmaceutical Technology, School of Pharmacy, International Medical University, Bukit Jalil, Kuala Lumpur 57000, Malaysia; HiraChoudhury@imu.edu.my (H.C.); ManishaPandey@imu.edu.my (M.P.)

**Keywords:** catalpol, type-1 diabetes mellitus, type-2 diabetes mellitus, diabetes complications

## Abstract

Catalpol isolated from *Rehmannia glutinosa* is a potent antioxidant and investigated against many disorders. This review appraises the key molecular pathways of catalpol against diabetes mellitus and its complications. Multiple search engines including Google Scholar, PubMed, and Science Direct were used to retrieve publications containing the keywords “Catalpol”, “Type 1 diabetes mellitus”, “Type 2 diabetes mellitus”, and “diabetic complications”. Catalpol promotes IRS-1/PI3K/AKT/GLUT2 activity and suppresses Phosphoenolpyruvate carboxykinase (PEPCK) and Glucose 6-phosphatase (G6Pase) expression in the liver. Catalpol induces myogenesis by increasing MyoD/MyoG/MHC expression and improves mitochondria function through the AMPK/PGC-1α/PPAR-γ and TFAM signaling in skeletal muscles. Catalpol downregulates the pro-inflammatory markers and upregulates the anti-inflammatory markers in adipose tissues. Catalpol exerts antioxidant properties through increasing superoxide dismutase (sod), catalase (cat), and glutathione peroxidase (gsh-px) activity in the pancreas and liver. Catalpol has been shown to have anti-oxidative, anti-inflammatory, anti-apoptosis, and anti-fibrosis properties that in turn bring beneficial effects in diabetic complications. Its nephroprotective effect is related to the modulation of the AGE/RAGE/NF-κB and TGF-β/smad2/3 pathways. Catalpol produces a neuroprotective effect by increasing the expression of protein Kinase-C (PKC) and Cav-1. Furthermore, catalpol exhibits a cardioprotective effect through the apelin/APJ and ROS/NF-κB/Neat1 pathway. Catalpol stimulates proliferation and differentiation of osteoblast cells in high glucose condition. Lastly, catalpol shows its potential in preventing neurodegeneration in the retina with NF-κB downregulation. Overall, catalpol exhibits numerous beneficial effects on diabetes mellitus and diabetic complications.

## 1. Introduction

According to the International Diabetic Federation (IDF), the global prevalence of diabetes is predicted to be approximately 463 million adults with over 1.1 million of them consisting of children and adolescents with type 1 diabetes mellitus (T1DM) [1]. Additionally, the data also showed that diabetes caused 4.2 million deaths in 2019, while 374 million people have a high risk of developing type 2 diabetes mellitus (T2DM). T1DM is a multifactorial disease and is caused by the autoimmune destruction of the β-cells in the islets of Langerhans. The pathogenesis of T2DM differs from T1DM as it is caused by a combination of insulin resistance and insulin deficiency. Individuals with risk factors such as genetic susceptibility, obesity, high-carbohydrate diet, or sedentary lifestyle are prone to insulin resistance. As the severity of insulin resistance progresses with the increasing of age and weight, individuals would proceed to develop impaired glucose tolerance or overt hyperglycaemia [2]. Diabetes mellitus (DM) is known to cause several complications. These complications can be categorized into microvascular and macrovascular. Microvascular complications include retinopathy, neuropathy, and nephropathy. Macrovascular complications include increased risk of stroke, cardiovascular diseases, and peripheral vascular diseases [3]. If diabetes and its complications are not managed well, it could often lead to death. Hence, finding a sustainable way to control diabetes and its complications is a matter of urgency. Based on the knowledge obtained from ancient systems including traditional Chinese medicine and Indian Ayurvedic medicine, there are probably more than 800 plants investigated in various experimental diabetic models. Some examples of traditional herbal medicines are *Gymnema sylvestre*, *Nigella sativa*, *Aloe vera*, *Camellia sinensis,* etc., and they are being used to treat or prevent the development of diabetes mellitus probably due to their strong antioxidant potential [4]. One such herb is *Rehmannia glutinosa*, which is originated from the northern and central parts of China. It has been used in traditional Chinese medicine to combat hyperglycemic symptoms for years [5]. Catalpol, an iridoid glucoside isolated from the root of this plant, has been reported as an active ingredient to produce the antidiabetic action, which is attributed to its potential antioxidant effect [6]. With years of investigation, it has been demonstrated that catalpol has acted through several pathways in managing T1DM, T2DM, and diabetic complications. However, the actions of catalpol are broad and require a detailed description to fully appreciate the roles of catalpol in diabetes. This review outlines the molecular and biochemical pathways of catalpol in alleviating DM and its complications.

## 2. Effects of Catalpol in Diabetes Mellitus

There are several in vivo and in vitro studies of catalpol that showed an improvement in the T1DM and T2DM through different molecular mechanisms. Catalpol (100 and 200 mg/kg, p.o., four weeks) administration significantly decreased the fasting blood glucose (FBG), serum insulin, homeostatic model assessment of insulin tolerance (HOMA-IR), total cholesterol (TC), triglycerides (TG), and low-density lipoprotein (LDL) and improved the oral glucose tolerance test (OGTT) and insulin tolerance test (ITT) in high-fat diet (HFD)/streptozotocin (STZ) induced diabetic mice [7,8,9,10,11]. In another study in HFD/STZ diabetic rats, catalpol (50 mg/kg, i.v., three weeks) treatment has shown a decreased plasma glucose by 66% and improved OGTT, body weight, and lipid profile [12]. In db/db mice, catalpol (160 mg/kg, p.o., four weeks) administration decreased the FBG and glycated serum protein (GSP) by 26% and 19%, respectively, with reduced serum insulin, HOMA-IR, TG, and TC and improved OGTT [13]. Further, catalpol (100–200 mg/kg, p.o., eight weeks) markedly reduced the FBG, decreased the GSP level, and improved the OGTT and ITT in db/db mice [14]. For T1DM, catalpol (0.1 mg/kg, i.v., 30 min) administration demonstrated a significant decrease (23%) in the blood glucose level in STZ induced diabetic rats [15]. Moreover, Wang et al. have reported that catalpol (50 and 100 mg/kg, p.o.) administration for four weeks decreased the FBG concentration in the STZ diabetic rats by 59% and 72%, respectively [16]. The molecular mechanism of catalpol for its antidiabetic activity is explained in detail with respect to its action on insulin-sensitive organs like the liver, skeletal muscle, adipose tissue, and pancreas.

### 2.1. Effects of Catalpol in the Liver

#### 2.1.1. Effect on Glucose Metabolism in the Liver

**Figure 1:**
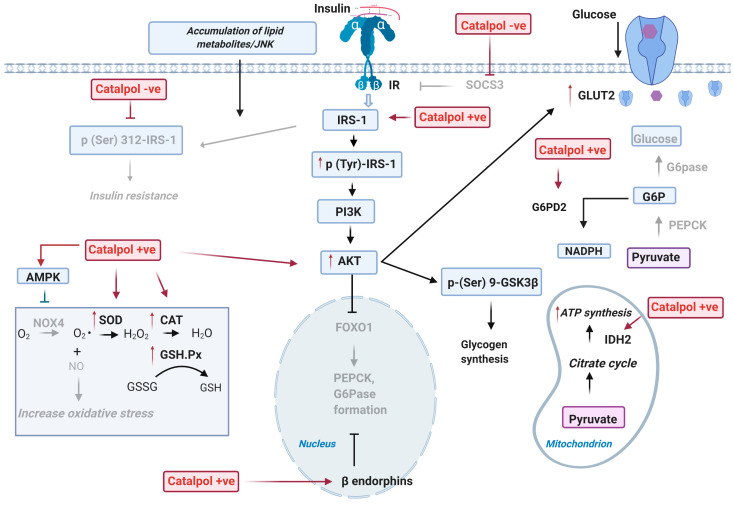
Anti-diabetic mechanism of catalpol in the liver. IR: Insulin Receptor, IRS-1: Insulin receptor substrate-1, p (Tyr)-IRS-1: Tyrosine phosphorylated insulin receptor substrate 1, p (Ser) 312-IRS-1: Serine 312-phosphorylated insulin receptor substrate 1, PI3K: Phosphoinositide 3-kinase, PDK1: Phosphoinositide-dependent kinase-1, PIP2: Phosphatidylinositol 4,5-bisphosphate, PIP3: phosphatidylinositol-3, 4, 5-triphosphate, AKT: Protein kinase B, GLUT4: glucose transporter type 4, GSK3β: Glycogen synthase kinase 3 beta, p-GSK3β: phosphorylation of glycogen synthase kinase 3 beta, FOXO1: forkhead box protein O1, p-FOXO1: Phosphorylation of forkhead box protein O1, PEPCK: Phosphoenolpyruvate carboxykinase, G6Pase: Glucose 6-phosphatase, HNF4: Hepatocyte nuclear factor 4, CRTC2: CREB Regulated Transcription Coactivator 2, AMPK: 5′ AMP-activated protein kinase, IDH2: Isocitrate dehydrogenase (NADP), G6P: Glucose 6-phosphate, G6PD2: Glucose-6-phosphate 1-dehydrogenase 2, NOX4: NADPH oxidase 4, NO: Nitric oxide, SOD: Superoxide dismutase, CAT: Catalase, GSH.Px: Glutathione peroxidase, GSSG: Glutathione disulphide, GSH: Glutathione, JNK: c-Jun N-terminal kinases, AdipoR1: Adiponectin receptor 1, SOCS3: Suppressor of cytokine signalling 3, ACC: Acetyl-coA carboxylase.

The liver is a vital organ that plays a central role in controlling glucose homeostasis. Insulin signaling in the liver is the key mechanism of glucose uptake and thus exhibiting glucose metabolism. Principal insulin signaling proteins such as insulin receptor substrate 1 (IRS-1), p-IRS-1, phosphatidylinositol-3-kinase (PI3K), protein kinase B (AKT), p-AKT, glycogen synthase kinase 3 beta (GSK3 β), and glucose transporter 2 (GLUT2) are widely studied to observe the change in insulin signaling in an insulin sensitive organ. Catalpol’s role in the liver of HFD/STZ induced type-2 diabetic model and db/db mice was studied. In the HFD/STZ mice model, catalpol (100 or 200 mg/kg, p.o., four weeks) reduced the p (Ser 307)-IRS-1 and increased the p (Ser 347)-AKT and p (Ser 9)-GSK3 β. The increase in p-AKT and p-GSK3 β and decrease of p-(Ser)-IRS1 alleviated the impaired insulin pathway in the liver through PI3K/AKT pathway (Figure 1) [17]. Furthermore, it is also proven that catalpol prevented gluconeogenesis by promoting the activation of AMP-activated protein kinase (AMPK), suppression of PEPCK, and G6Pase protein expression. Thus, these results indicated that catalpol promoted glycogenesis and suppressed gluconeogenesis by activating the PI3K/AKT pathway in HFD/STZ mice [7]. Bao at al. showed that p-AMPK and GLUT protein expression were suppressed in db/db mice. Catalpol (80 or 160 mg/kg, p.o., four weeks) treatment significantly increased p-AMPK and GLUT protein expression in liver, skeletal muscle, and adipose tissue, which facilitates the glucose uptake into the cells [13]. Liu et al. demonstrated that the expression of IRS-1, isocitrate dehydrogenase 2 (IDH2), and glucose-6-phosphate 1-dehydrogenase 2 (G6PD2) were downregulated, and the suppressor of cytokine signaling 3 (SOCS3) expression was upregulated in db/db mice due to insulin resistance [14]. The lowered expression of IRS-1 resulted in negative regulation of insulin signaling cascades, as IRS-1 is an important ligand in activating the PI3K/AKT pathway [17]. IDH2, an enzyme that catalyzes the citrate cycle and decreased IDH2 activity eventually attenuates glucose metabolism and ATP production [18]. Moreover, G6PD2 is an enzyme that catalyzes the pentose phosphate pathway that utilizes glucose to produce NADPH and ribose-5-phosphate. The downregulation of G6PD2 enzyme decreases the glucose metabolism [19]. Nevertheless, SOCS3 is an enzyme that inhibits the tyrosine phosphorylation of insulin receptor. Therefore, SOCS3 up-regulation results in the suppression of insulin signaling pathway [20]. It was reported that catalpol (100 or 200 mg/kg, p.o., eight weeks) significantly reversed the IRS-1, IDH2, and G6PD2 downregulation and decreased the expression of SOCS3 in db/db mice. Thus, the results imply that catalpol could increase glucose metabolism through accelerating the citrate cycle and pentose phosphate pathway and promoting insulin signaling pathway [14]. In an in vitro study, human hepatocellular carcinoma (HepG2) cell line was administrated with glucosamine to induce insulin resistance [21]. In glucosamine-treated HepG2 cells, p-AKT, Ser (256) phosphorylation of forkhead box protein O1 (p (Ser 256)-FOXO1), and AMPK levels were significantly decreased suggesting extensive gluconeogenesis. Contrarily, catalpol (20–80 µM) administration increased the p-AKT, p (Ser 256)-FOXO1 level, and p-AMPK in glucosamine-treated HepG2 cells. Therefore, these results showed that catalpol activated glycogenesis through PI3K/AKT pathway and suppressed glucosamine-induced gluconeogenesis by downregulating enzymes involved in gluconeogenesis [7].

In STZ-induced T1DM rats, catalpol (0.1 mg/kg, i.v.) administration significantly controlled the glycemic parameters. These effects were correlated with an enhanced β-endorphins secretion from the isolated adrenal medulla of the rats [15]. β-endorphins is an endogenous opioid neuropeptide that exerts its effects such as analgesic when it binds to the opioid µ-receptors [22]. Evidently, other than analgesic effect, activation of opioid µ-receptor by β-endorphins also improved glucose homeostasis through the downregulation of PEPCK expression, which is a rate-limiting enzyme in gluconeogenesis [23]. Thus, catalpol suppresses gluconeogenesis through increased β-endorphins secretion and activation of opioid µ-receptors in STZ-diabetic rats [15]. Thereby, catalpol improved glucose uptake and glucose metabolism in the liver of diabetic state [13].

#### 2.1.2. Effect on Oxidative Stress in the Liver

Oxidative stress plays an important role in leading to insulin resistance [24]. The NADPH oxidative 4 (NOX4) is an enzyme that catalyzes the formation of ROS and leads to oxidative damage in the insulin-sensitive cells [25]. Another enzyme, malondialdehyde (MDA), is a marker of lipid peroxidation [26]. The key findings by Yan et al. showed that MDA and NOX4 levels were increased, whereas the endogenous antioxidants such as SOD and GSH-Px were decreased in HFD/STZ diabetes mice, which suggested an elevated oxidative status. In contrast, catalpol (100 or 200 mg/kg, p.o., four weeks) treatment significantly reversed the reduced antioxidant enzyme (SOD and GSH-Px) level and suppressed the serum level of MDA and NOX4 in HFD/STZ mice. These results proved that catalpol alleviated oxidative stress through upregulation of antioxidant enzyme level and downregulation of NOX4 protein in T2DM mice [7]. In glucosamine-treated HepG2 cells, catalpol (20–80 µM) significantly increased the SOD and GSH-Px level and decreased the MDA level and NOX4 protein expression in HepG2 cells. Ultimately, catalpol also suppressed the oxidative stress induced by glucosamine in HepG2 cells [7]. A high glucose exposure to HepG2 cells caused a marked increase of ROS production in the cells. Catalpol (50 µM) treatment efficiently suppressed the ROS formation by upregulating the superoxide dismutase 2 (SOD2) expression [8]. SOD2 is an enzyme that binds to the superoxide byproducts of oxidative phosphorylation to prevent ROS production in the mitochondria [27]. Thus, catalpol showed a defensive effect against the oxidative attack of the cell through the increased of SOD2 level.

Moreover, catalpol also enhanced the mitochondria antioxidative capacity in HFD/STZ-diabetic mice. The manganese-dependent superoxide dismutase (MnSOD) is an antioxidative enzyme presents in the mitochondria matrix of liver tissue, used to catalyze the reactive oxygen species production [28]. After catalpol (100 or 200 mg/kg, p.o.) treatment, the activity of MnSOD was significantly promoted, which showed that catalpol also improved antioxidative capacity in the liver tissue through increased MnSOD [8]. Evidently, the studies on HFD/STZ induced diabetic mice were reported to have fewer mitochondria in liver. This is associated with the decrease of mitofusin-1 (Mfn-1), the increase of dynamin-1-like protein (Drp 1), and mitochondria fission protein-1 (Fis-1). Both dysregulations eventually attenuated the ATP production, as Mfn-1 is a mediator for mitochondria fusion, whereas Fis-1 and Drp-1 are proteins encoded to promote mitochondria membrane fragmentation [29,30]. Xu et al. has reported that catalpol (100 or 200 mg/kg, p.o.) upregulated the expression of Mfn-1 and downregulated the Fis 1 and Drp-1 protein through an in vivo study in HFD/STZ mice. In high glucose treated HepG2 cells, the mitochondria ATP content and membrane potential were significantly reduced in the high glucose treated HepG2 cells. Catalpol (50 µM) administration significantly restored the ATP content and membrane potential in the cells, and these are correlated with the increase of Mfn1 expressions and decrease of Fis-1 and Drp-1 of proteins involved in mitochondria fusion and fission, respectively. Thus, catalpol upregulated mitochondria fusion and downregulated mitochondria fission through increase of Mfn-1 and decrease of Fis-1/Drp1, respectively, thereby causing an increased mitochondria synthesis in HepG2 cells [8]. Therefore, the treatment with catalpol effectively increased mitochondria formation and reduction in oxidative stress in the liver.

#### 2.1.3. Effect on Lipid Metabolism in the Liver

Uncontrolled lipogenesis is considered as one of the risk factors in DM [31]. Acetyl-CoA carboxylase (ACC) and 3-Hydroxy-3-Methylglutaryl-CoA Reductase (HMGCR) are the key enzymes in lipogenesis, as they regulate TG and TC synthesis, respectively [32]. ACC and HMGCR level was upregulated in the liver cells of db/db mice. Moreover, there is substantial evidence that suggests that hyperlipemia results in impaired insulin signaling pathway, as high TG/TC suppresses glucose uptake and promotes gluconeogenesis in the liver [33]. Therefore, the lowered ACC and HMGCR level would improve the impaired insulin signaling pathway. Catalpol (80 or 160 mg/kg, p.o., four weeks) significantly downregulated the mRNA expression of ACC and HMGCR in db/db mice. Thus, it was suggested that catalpol improved glycemic control in db/db mice by inhibiting lipogenesis [13]. PPAR-γ is crucial in regulating triglycerides homeostasis and protecting other tissues from insulin resistance by repartitioning the fatty acid to adipocyte [34]. Yap et al. showed that the PPAR-γ gene and protein expression was declined in the T2DM mice. The decreased PPAR-γ gene expression resulted in increased triglycerides level and insulin resistance in the liver. After catalpol (200 mg/kg, p.o.) treatment, the expression of PPAR-γ gene increased and a significant reduction of FBG, HOMA-IR, and liver TG was reported. Moreover, the liver hepatocyte and glycogen content were reversed by catalpol in T2DM mice. Therefore, these results indicate that catalpol significantly enhanced insulin signaling and suppressed lipogenesis in the liver by increasing PPAR-γ expression [35].

The underlying mechanisms of catalpol in the liver include an increasing insulin signaling (p-IRS-1, p-AKT, p-GSK3β p-AMPK, GLUT2) and β-endorphins and decreasing p-FOXO1 protein, PEPCK, and G6Pase expression [7,13,15]. Additionally, catalpol also increases glucose metabolism and decreases lipogenesis within the liver by increasing IDH-2, G6PD2, and adiponectin and decreasing ACC, HMGCR, and SOCS3 expression [13,14]. In addition, catalpol reduced oxidative stress through increasing SOD and GSH levels, decreasing MDA and NOX4 activity [7]. Catalpol improves mitochondria synthesis through upregulating Mfn1 expression and MnSOD and SOD2 activity and downregulating Fis-1 and Drp-1 expression [8]. Thus, catalpol treatment was found to significantly improve metabolic function in the diabetic liver through the pathways as described in Figure 1.

### 2.2. Effects of Catalpol in the Skeletal Muscle

**Figure 2:**
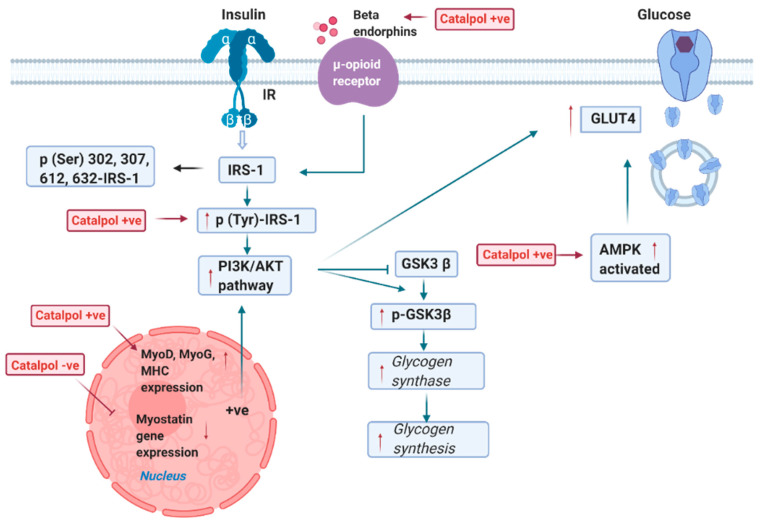
Antidiabetic mechanism of catalpol in the skeletal muscle. MOR: µ-opioid receptor, IR: Insulin receptor, GLUT4: Glucose transporter type 4, IRS-1: Insulin receptor substrate-1, p (Tyr)-IRS-1: Tyrosine phosphorylated insulin receptor-1, p (Ser) 302, 307, 612, 632-IRS-1: Serine 302, 307, 612, 632-phosphorylated insulin receptor substrate-1, PI3K/AKT: Phosphoinositide 3-kinase/Protein kinase B pathway, GSK3β: glycogen synthase kinase 3 beta, p-Gsk3β: phosphorylation of glycogen synthase kinase 3 beta, AMPK: 5′ AMP-activated protein kinase, p-TBC1D4: phosphorylation of TBC1 Domain Family Member 4, Rab-GDP:, Rab-GTP: Ras-associated binding- guanosine triphosphate, Rab-GDP: Ras-associated binding-guanosine diphosphate, MyoD: myoblast determination protein 1, MyoG: Myogenin, MHC: Myosin heavy chain.

Skeletal muscle is the predominant site for glucose utilization, as it is the largest organ involved in glucose homeostasis [36]. Catalpol was studied in pre-diabetes mice, where C57BL6/J mice were fed with HFD [9]. Xu et al. demonstrated that after eight weeks of HFD, the protein levels of p-IRS-1, p-AKT, and GLUT4 were significantly lower in the skeletal muscle of pre-diabetic mice. As p-IRS-1 stimulates the activation of p-AKT and translocation of GLUT4 to subsequent glucose uptake, the lowered level of these proteins could lead to impaired glucose uptake (Figure 2) [17]. In contrast, catalpol (200 mg/kg, p.o., 4–8 weeks) administration reversed the suppression of p-IRS-1, p-AKT, and GLUT4 protein levels in the skeletal muscle of pre-diabetic mice. Thereby, catalpol significantly increased the skeletal muscle insulin sensitivity by activating IRS-1/AKT/GLUT4 in the skeletal muscle of pre-diabetic mice [9].

The db/db mice displayed a decline in muscle grip strength and skeletal muscle weight, which correlated with the decrease of myoblast determination protein 1 (MyoD), myogenin (MyoG), and myosin heavy chain (MHC) gene expression and the increase of myostatin expression [37]. MyoD and MyoG are the muscle-specific transcription factors found in the satellite cells (muscle stem cells) and responsible for differentiating myotubes from satellite cells, which is known as myogenesis [38]. Moreover, MHC is the muscle thick filament protein that enhances the terminal differentiation of satellite cells [39]. Further, myostatin is a protein released by myocytes to inhibit myogenesis [40]. The suppression of MyoG, MyoD, and MHC and increase of myostatin gene could result in a decreased myogenesis and attenuation of glucose uptake into the muscle cells. Catalpol (200 mg/kg, p.o., eight weeks) upregulated the MyoD, MyoG, and MHC expression and downregulated the myostatin expression in db/db mice. Therefore, catalpol enhances glucose metabolism by activating myogenesis in db/db mice [37]. Bao et al. showed that p-AMPK and GLUT4 level were significantly reduced in the skeletal muscle of db/db mice. The activated AMPK is important to increase the phosphorylation of TBC1 Domain Family Member 4 (TBC1D4), which is a Rab-GTPase-activating protein that accelerates glucose homeostasis by regulating the translocation of GLUT4 [41]. The decrease of p-AMPK would lower the phosphorylation of TBC1D4, ultimately suppressing GLUT4 translocation [42]. Catalpol (80 or 160 mg/kg, p.o., four weeks) significantly increased p-AMPK and GLUT4 proteins in the skeletal muscle, which promoted glucose uptake [13].

In STZ-induced T1DM mice, Shieh et al. demonstrated that GLUT4 protein expression was downregulated in the skeletal muscle. In contrast, catalpol (0.1 mg, i.v.) significantly increased GLUT4 expression in T1DM rats by enhancing the secretion of β endorphins from adrenal medulla [15]. C2C12 cells are the mouse myoblast cell line that differentiates rapidly to form myotubes [43]. C2C12 cells were supplemented with 50 mM glucose to induce high glucose damage in the cells, and it showed that p-IRS-1, PI3K, p-AKT, and GLUT4 protein levels were significantly decreased, whereas catalpol (10, 30, 100 µM) administration significantly augmented the p-IRS-1, p-AKT, PI3K, and GLUT4 protein expression in high glucose C2C12 cells (Figure 2). Thus, catalpol could stimulate glucose homeostasis through the PI3K/AKT insulin signaling pathway in C2C12 cells. Moreover, catalpol (10, 30, 100 µM, 24 h) administration increased MyoD and MyoG mRNA/protein levels in high glucose treated C2C12 cells. Therefore, catalpol also enhanced myogenesis in C2C12 cells (Figure 2) [37]. To explore whether myogenesis is critical to activate PI3K/AKT insulin signaling pathway, small interfering RNA (siRNA) was used to knockdown MyoD and MyoG in C2C12 cells. As expected, catalpol induced MyoD1 and MyoG expression were downregulated in siRNA treated C2C12 cells. Moreover, after catalpol treatment, p-IRS-1, p-AKT, PI3K, and GLUT4 protein levels remained lowered by siRNA-MyoD and siRNA-MyoG. Thus, these results indicate that the catalpol could induce PI3K/AKT insulin signaling by enhancing MyoD/MyoG expression [37].

**Figure 3:**
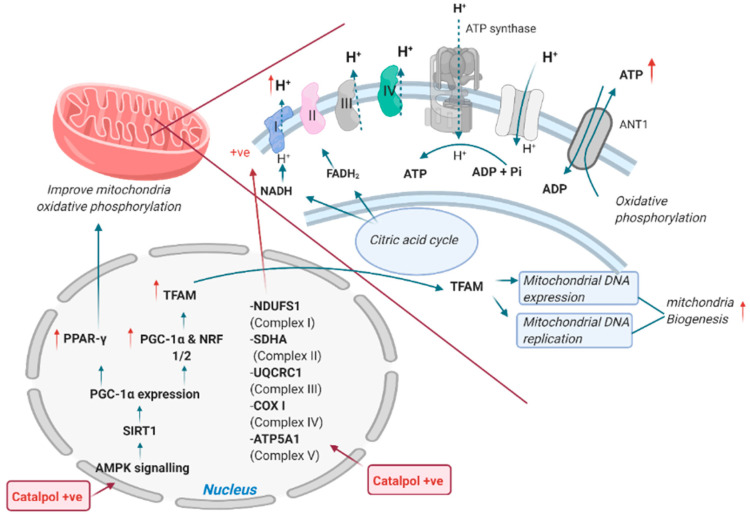
Effect of catalpol in the skeletal muscle mitochondria for alleviating diabetes mellitus. AMPK: 5′ AMP-activated protein kinase, SIRT1: Sirtuin-1, PGC-1α: Peroxisome proliferator-activated receptor gamma coactivator 1-alpha, PPAR-γ: Peroxisome proliferator-activated receptor gamma, NRF-1/2: Nuclear respiratory factor-1/2, TFAM: mitochondrial transcription factor A, NDUFS1: NADH-ubiquinone oxidoreductase, SDHA: Succinate dehydrogenase, UQCRC1:Ubiquinol-cytochrome c reductase core protein 1, COX1: Cytochrome c oxidase subunit 1, ATP5A1: ATP synthase F1 subunit α, NADH: Reduced nicotinamide adenine dinucleotide, FADH2: Reduced flavin adenine dinucleotide, ATP: Adenosine triphosphate, ADP: Adenosine diphosphate, ANT1: Adenosine nucleotide transporter 1.

Mitochondria serve to regulate glucose by producing energy in skeletal muscle cells [44]. Declined mitochondria membrane potential, ATP, and mitochondria biogenesis in skeletal muscle of db/db mice were reported, which were associated with decreased p-AMPK, PGC-1α, and TFAM (transcription factor A, mitochondrial) expression. PGC-1α expression is mainly regulated by protein kinases (such as AMPK and AKT) and SIRT1, a protein deacetylase. AKT reduced PGC-1α expression through inducing the serine phosphorylation of PGC-1α, which decreases FOXO1 activity, blunting gluconeogenesis in liver [45]. In skeletal muscle, the activated AMPK induced the activation of SIRT1 through an indirect increase in cellular NAD+ levels. Then, SIRT1 enhanced PGC-1α transcriptional activation leading a markedly PGC-1α activity. PGC-1α is a master regulator of mitochondrial biogenesis through activation of TFAM and replication of mitochondria DNA (Figure 3) [42]. However, the suppressed AMPK/PGC-1α/TFAM pathway can lead to decreased mitochondrial number, which will eventually downregulate the ATP formation. Xu et al. showed that catalpol (200 mg/kg, p.o., eight weeks) significantly enhanced the p-AMPK, PGC-1α, and TFAM in the skeletal muscle in db/db mice. It was postulated that hypoglycemic effect of catalpol is due to the enhancement of mitochondria biogenesis through AMPK/PGC-1α/TFAM signaling [46]. Oxidative phosphorylation is important in the regulation of mitochondria membrane potential and ATP synthesis. In T2DM in vivo models, the protein expression of mitochondria membrane complexes such as NDUFS1 (Complex 1), SDHA (Complex II), UQCRC1 (Complex 3), COX1 (Complex IV), and ATP5A1 (Complex V) was markedly decreased in the skeletal muscle. Mitochondria membrane complexes are important in oxidative phosphorylation, as these complexes set-up a proton concentration gradient and allow the formation of ATP when the proton passes into the mitochondria matrix through Complex V (Figure 3) [47]. As a result, the decreased mitochondria membrane complexes dysregulated the membrane potential and ATP synthesis. Catalpol (200 mg/kg, p.o., eight weeks) increased the mitochondria membrane complexes in T2DM experimental models. Therefore, catalpol has shown the anti-hyperglycemic activity through the improved mitochondrial membrane potential and ATP synthesis mediated by the enhanced protein expression of mitochondria complexes [9,10,46]. Moreover, Yap et al. showed that catalpol (200 mg/kg, p.o., four weeks) enhanced mitochondria respiration through the upregulated expression of PPAR-γ expression in HFD/STZ model [48]. PPAR-γ expression is activated by PGC-1α; thus, the upregulation of PGC-1α increases PPAR-γ expression. PPAR-γ is a ligand-activated transcription factor s present with a low level in the skeletal muscle but has a wide spectrum in regulating mitochondria function. The activated PPAR-γ enhances oxidative phosphorylation by preventing the loss of mitochondria membrane potential and increasing mitochondria DNA production [49]. Moreover, constitutive activation of PPAR-γ in the skeletal muscle attenuates intramuscular lipid accumulation and protects against susceptibility to diet-induced insulin resistance [50]. Therefore, the results suggested that catalpol improved mitochondria function through upregulating PPAR-γ expression. Collectively, catalpol enhanced glucose utilization through increasing p-IRS, p-AKT, p-AMPK, GLUT4, and β-endorphins and promoted myogenesis through upregulating MyoD, MyoG, and MHC expression and decreasing myostatin expression in skeletal muscle [9,15,37]. In addition, catalpol improved muscle mitochondria biogenesis and function through AMPK/PGC-1α/PPAR-γ/TFAM signaling [10,46] and insulin sensitivity and mitochondrial respiration through AMPK/SIRT1/PGC-1α/PPAR-γ activation [48].

### 2.3. Effects of Catalpol in the Adipose Tissue

Adipose tissues contribute to systemic inflammation by producing extensive varieties of adipokines, cytokines, and chemokines [51]. In obese individuals, the adipose tissue expansion is correlated to the accumulation of macrophages and other inflammatory cells such as mast cells or neutrophils [51]. Macrophage polarization is critical for inflammation and tissue homeostasis [52]. The macrophage can be polarized into two distinct forms, known as M1 or M2. M1 is the classically activated macrophage that promotes the secretion of M1-proinflammatory factors such as TNF-α, IL-6, IL-1β, MCP-1, iNOS, and CD11C to induce inflammation, whereas M2, the alternatively activated macrophage, induces high levels of anti-inflammatory factor such as arginase-1, Ym-1, IL-10, MGL1, Clec7a, and MMR to counter the inflammatory response [53]. In HFD induced T2DM mice, Zhou et al. demonstrated that the expression of M1-proinflammatory factors markedly increased and M2-anti-inflammatory factors were decreased in adipose tissue [11]. Evidently, the markedly upregulated M1 pro-inflammatory factor has been shown to prevent insulin action in insulin-responsive cells through autocrine and paracrine mechanisms [54]. On the contrary, catalpol (100 mg/kg, p.o., four weeks) significantly downregulated the M1-pro-inflammatory expression level and upregulated the M2- anti-inflammatory expression in HFD-diabetic mice, thereby decreasing insulin resistance through the suppression of inflammation [11]. Additionally, the p-JNK, p-IKκβ, and the activation of NF-κβ were significantly enhanced in the adipose tissue of HFD diabetic mice [11]. Many researchers have identified that the phosphorylated JNK negatively regulates the insulin signaling pathway and simultaneously leads to insulin resistance [55,56]. Additionally, IKκβ is a kinase that is involved in propagating the cellular inflammatory response through activation of NF-κβ, a family of transcription factors that stimulate the transcription of inflammatory genes [55,57]. Therefore, the inhibition of p-JNK, p-IKκβ, and NF-κβ could counteract insulin resistance in HFD-diabetic mice. In this regard, catalpol (100 mg/kg, p.o., four weeks) has been reported to suppress the p-JNK, p-IKκβ, and the activation of NF-κβ in the adipose tissue of HFD-diabetic mice. Collectively, these results imply that catalpol treatment could ameliorate insulin resistance in T2DM mice through attenuating the inflammatory protein signaling in the adipose tissues [11]. Oxidative stress and a high level of advanced glycation end products (AGEs) have been known as the key underlying cause of the development of DM and its complications [58]. AGEs are a diverse group of highly oxidant compounds that bind to their receptor (RAGE) to produce ROS and pro-inflammatory molecules [59]. Catalpol (500 µM) suppressed the ROS production in AGE-treated THP-1 cells, which mimic the situation of inflamed adipose tissue. Other than that, catalpol (500 µM) also downregulated the expression RAGE in AGE-treated THP-1 cells. Catalpol (500 µM) also inhibited NOX4 activity in THP-1 cells to prevent ROS formation. Overall, catalpol could inhibit oxidative stress through the suppression of AGE-induced ROS production and NOX4 activity [60]. Catalpol has been reported to elevate the plasma adiponectin level in db/db mice. Adiponectin released from adipose tissue exerts its action when it binds to its receptors (AdipoR1 or AdipoR2), and the function of adiponectin includes activating AMPK to increase glucose utilization [61]. Due to insulin resistance or inflammation, the serum adiponectin level was decreased in db/db mice, which were reversed by catalpol (80 or 160 mg/kg, p.o., four weeks) treatment [13].

**Figure 4:**
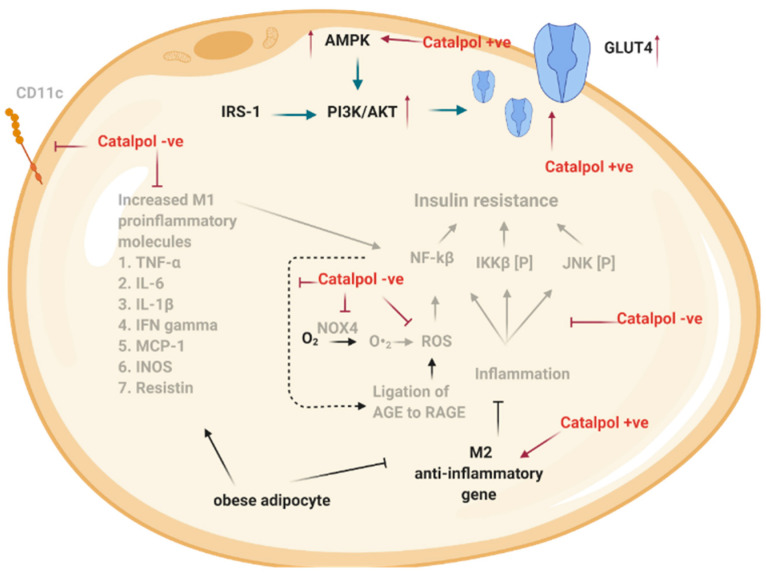
Antidiabetic mechanism of catalpol in the adipocyte. IRS-1: Insulin receptor substrate-1, PI3K/AKT: Phosphoinositide 3-kinase/Protein kinase B pathway, AMPK: 5′ AMP-activated protein kinase, GLUT4: Glucose transporter type 4, JNK: c-Jun NH2-terminal kinase, IKκβ: Inhibitor of κβ kinase, NF- κβ: Nuclear factor κ-light-chain-enhancer of activated β cells, NOX4: NADPH oxidase 4, ROS: Reactive oxygen species, AGE: Advanced glycation end-products, RAGE: Receptor of advanced glycation end-products, TNF-α: Tumour Necrosis Factor alpha, IL-6: Interleukin-6, IL-1β: Interleukin 1 beta, IFN gamma: Interferon gamma, MCP-1: Monocyte chemoattractant protein-1, INOS: Inducible nitric oxide synthase, CD11C: Integrin.

In summary, catalpol exhibits anti-inflammation in adipose tissue through suppressing M1-pro-inflammatory factors (TNF-α, IL-6, IL-1β, MCP-1, iNOS, and CD11C), increasing M2-anti-inflammatory factors (arginase 1, Ym-1, IL-10, MGL1, Clec7a, and MMR) and decreasing p-JNK, p-IKκβ, and NF-κβ activation (Figure 4) [11]. Further, catalpol suppressed ROS formation through decreasing RAGE expression and NOX4 activity [60].

### 2.4. Effects of Catalpol in the Pancreas

**Figure 5:**
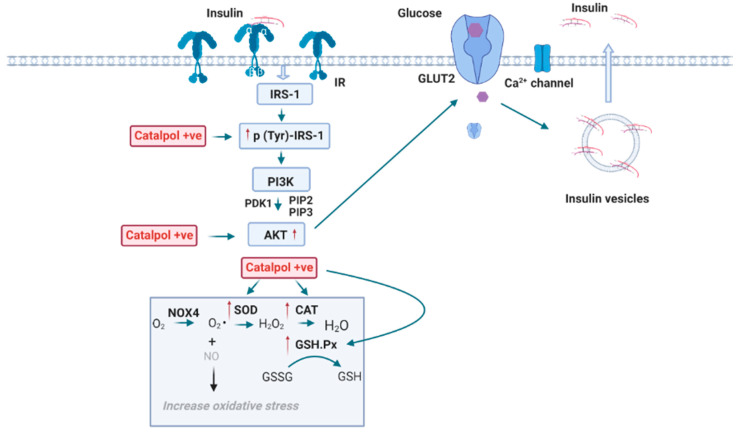
Antidiabetic mechanism of catalpol in the pancreatic β-cell. IR: Insulin Receptor, IRS-1: Insulin receptor substrate-1, p (Tyr)-IRS-1: Tyrosine phosphorylated insulin receptor substrate-1, PI3K: Phosphoinositide 3-kinase, PDK1: Phosphoinositide-dependent kinase-1, AKT: Protein kinase B, GLUT2: glucose transporter type 2, NOX4: NADPH oxidase 4, NO: Nitric oxide, SOD: Superoxide dismutase, CAT: Catalase, GSH.Px: Glutathione peroxidase, GSSG: Glutathione disulphide, GSH: Glutathione.

The autocrine action of insulin plays a remarkable role in β-cells function. Insulin controls its secretion and islet cell differentiation by positively regulating the insulin signaling cascade [62]. Recently, Elhassan et al. reported that p-IRS, PI3K, p-AKT, and AKT proteins in high glucose (HG) treated INS-1E pancreatic β-cells were downregulated. Thereby, the suppression of p-IRS, PI3K, p-AKT, and AKT proteins indicated that a HG environment has impaired the autocrine action of insulin. On the contrary, catalpol (30 mM) significantly increased the p-IRS, PI3K, p-AKT, and AKT proteins in HG-treated INS-1E cells. Thus, catalpol could ameliorate insulin sensitivity through the insulin-signaling pathway in β-cells [63,64]. Moreover, catalpol may act against T2DM-induced oxidative damage in pancreatic islet cells of HFD/STZ rats through antioxidant effects. Zhu et al. showed that the endogenous antioxidants such as SOD, GSH-Px, and CAT decreased, and the MDA levels increased, which indicates that oxidative stress was strengthened in HFD/STZ diabetic rats. Catalpol (50 mg/kg, i.v., two weeks) significantly improved SOD, GSH-Px, and CAT levels and reduced the MDA level in HFD/STZ diabetic rats. Therefore, catalpol could reduce oxidative stress by recovering the balance of endogenous oxidants and antioxidants [12]. In brief, catalpol improves the autocrine action of insulin through upregulating the IRS-PI3K-Akt signaling and attenuating oxidative stress in pancreatic β-cells (Figure 5). However, the effect of catalpol on the autocrine action of insulin could be further supported by in vivo studies.

## 3. Effects of Catalpol in Diabetes Complications

### 3.1. Effects in Diabetic Nephropathy

**Figure 6:**
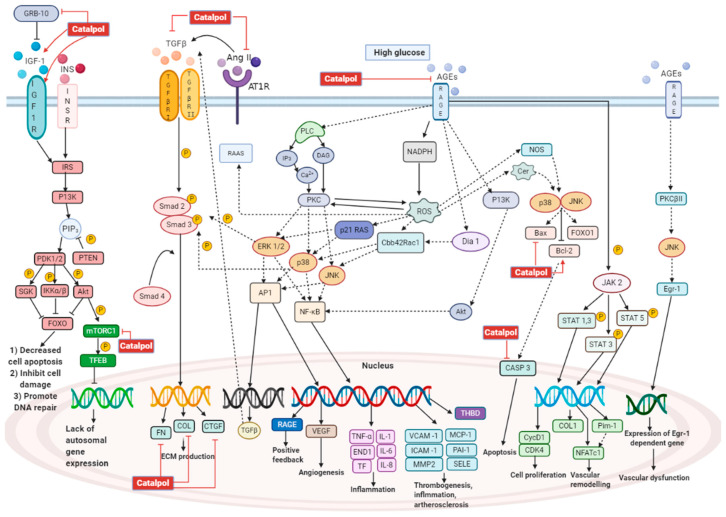
Molecular mechanism of catalpol in diabetic nephropathy. Under high glucose, AGE/RAGE pathway is activated, leading to angiogenesis, inflammation, cell apoptosis, vascular remodeling, and vascular dysfunction. AGE/RAGE pathway is able to stimulate the renin–angiotensin–aldosterone system (RAAS) and lead to production of angiotensinogen II (Ang II). AGE/RAGE pathway could also stimulate the release of TGF-β. Both Ang II and AGE/RAGE increase the concentration of TGF-β, which increases the production of extracellular matrix (ECM). In DM, insulin signaling pathway is disrupted, which leads to the impairment of downstream pathway for protein kinase B (Akt) and Forkhead box protein O1 (FOXO1). Akt pathway is responsible for inhibiting apoptosis, while FOXO1 pathway plays a significant role in oxidative stress resistance and DNA repair. Hence, impairment of insulin signaling pathway leads to increased cell apoptosis, cell damage, and lack of DNA repair. In DM, mTORC1 is activated, which deactivates transcription factor EB (TFEB). This causes lack of autosomal gene expression. Catalpol demonstrated nephroprotective effect by deactivating AGE/RAGE and its downstream pathway. Catalpol could also decrease the production of TGF-β and Ang II, whereas it stimulates the insulin signaling pathway by deactivating GRB-10 and activating IGF-1/IGF-1R. Catalpol also increases the autophagy through deactivating mTORC1 and activating TFEB proteins.

Globally, diabetes has caused more than 80% of end-stage renal disease (ESRD). People with diabetic nephropathy (DN) have 10 times higher prevalence to have ESRD compared to those without DN [1]. Hyperglycemia is able to cause morphological changes in kidney and eventually lead to decreased kidney function. DN is characterized by albuminuria, increased serum creatinine level, increased blood urea nitrogen level, and declined estimated glomerular filtration rate (eGFR). The pathophysiology of DN is due to glomerulus sclerosis, expansion of mesangial cells, and damage on podocytes [65]. In DM, hyperglycemia is able to alter the homeostasis of endothelial cells in the vascular wall, which later leads to the production of glucose by-products and metabolites that are known to initiate DN [65]. Excess glucose within the endothelial cells favors the formation of AGE and stimulates the AGE/RAGE pathway [66]. Through this pathway, PKC activation leads to vascular endothelial growth factor (VEGF) production and increased permeability of the endothelium. Thus, hyperfiltration occurs and albumin could pass through the glomerulus filtration barrier easily, leading to albuminuria [66]. Under high glucose levels, ROS is formed through the electron leakage in complex I of mitochondria, leading to the formation of superoxide (O2−) [67]. ROS stimulates the AGE formation activating the AGE/RAGE pathway [66]; NADPH oxidase (NOX) is upregulated through AGE/RAGE pathway, causing more ROS production and oxidative stress [66]. An increase in oxidative stress disrupts the formation of nitric oxide (NO), causing endothelial dysfunction [65]. The formation of NO is further being suppressed by O2−, as they could react together and form peroxynitrite (ONOO−). ONOO− is able to oxidize protein and DNA, causing cell apoptosis, inflammation, and mitochondrial dysfunction [68]. Inflammatory cytokines such as vascular cell adhesion protein 1 (VCAM-1), E-selectin, and intercellular adhesion molecule 1 (ICAM-1) are recruited through the AGE/RAGE pathway [68]. Under hyperglycemia, renin-angiotensinogen-aldosterone-system (RAAS) is activated, and angiotensinogen II (Ang II) causes intraglomerular hypertension. This increased pressure induces hyperfiltration and increases the eGFR. Intraglomerular hypertension is also known to cause glomerular sclerosis [65]. Transforming growth factor-β (TGF-β), a pro-fibrotic factor, is being upregulated through the AGE/RAGE pathway and RAAS. TGF-β activation via smad2/3 pathway causes accumulation of extracellular matrix (ECM). ECM proteins such as fibronectin (FN) and collagen are being synthesized and deposited within the interstitial space of mesangial cells, as seen in glomerulosclerosis and mesangial cell expansion [65]. Under physiological conditions, the insulin signaling pathway prevents cell apoptosis by activating PI3K/AKT signaling, which is generally disrupted in DN [69]. Insulin resistance has caused dysfunction in the insulin signaling, which promotes cell apoptosis. In DN, mTORC1 is activated, which deactivates transcription factor EB (TFEB), causing the attenuation of autosomal gene expression and decrease in autophagy [70]. Catalpol has demonstrated the protective effect on the kidney via several pathways, as shown in Figure 6.

In vivo studies of catalpol have been reported in several DN models. Jiang et al. studied the activity of catalpol (1 g/kg, p.o., 16 weeks) on DN in db/db mice [71]. Yang et al. have induced DN in C57BL/6 mice by using STZ, and catalpol was administered (10 mg/kg/d, i.p., 14 days) [72]. Catalpol (30, 60, and 120 mg/kg, p.o., 10 weeks) was administered to male Sprague–Dawley (SD) DN rats induced with HFD and STZ injection [73]. Chen et al. have administered catalpol (50 and 100 mg/kg/d, p.o., eight weeks) to DN mice induced by HFD fed in male KK-Ay mice [74]. Male C57BL/6J mice were given STZ injection to induce DM, and catalpol (30, 60, and 120 mg/kg, p.o., four weeks) was administered to these DM mice [75]. In another study, catalpol (100 and 200 mg/kg, p.o., 28 days) was given to HFD/STZ induced DN in C57BL/6 mice [76]. The findings of these studies suggested that catalpol attenuated the morphological changes in the kidney due to DN. By using Periodic Acid–Schiff staining, glycoproteins in the kidney were stained in purple color, which is more severe in DN, whereas catalpol treatment reduced the glycoprotein deposition [71,72,73,74,75,76]. This indicates that catalpol is able to ameliorate kidney injury by preventing the deposition of glycoprotein. Masson staining is utilized to stain the collagen fibers within the kidney cells, which is more intense (blue color) in DN kidneys. The intensity was markedly reduced in catalpol treated animals, suggesting the alleviation of fibrosis [71,72,76]. Further, hematoxylin and eosin (H&E) staining in DN group demonstrated an expansion in the glomerular cavity, increased inflammatory cells in the interstitium, glomerular atrophy, disarranged renal tubules, and vacuolar lesion in the epithelial cells. All these changes in the kidney were significantly reduced by catalpol [76]. Catalpol has demonstrated a reduced thickening and matrix deposition in the glomerular basement membrane of the DN group observed microscopically using Periodic Schiff–Methenamine Silver (PASM) staining [76]. By using TUNEL assay together with DAPI staining, the cell viability in kidney and podocyte mitochondria is observed. In DN, kidney cells together with podocyte mitochondria showed large portion of cell death. Catalpol at 1, 5, and 10 µM concentrations significantly reduced cell death and increased cell viability [76]. The ultrastructure of podocytes in DN is shown to have a bigger gap and smaller density [75] and swollen, vacuole lesion and rupture of the outer membrane of podocyte mitochondria in DN kidneys [74], whereas catalpol significantly prevented these changes in podocytes. By using JC-1 staining, the mitochondrial membrane potential can be analyzed. The normal membrane potential is stained in red, while alteration in membrane potential is stained in green. In DN, the proportion of red-stained is less and the green stain is increased compared to normal control. However, catalpol of 5 and 10 µM significantly increased the red proportion and decreased the green proportion [76]. Catalpol can reduce fibrosis in the kidney by decreasing the accumulation of ECM. Dong and Chen have proven that catalpol (60 and 120 mg/kg; p.o.) administration in male SD rats showed decreased Ang II, TGF-β1, and connective tissue growth factor (CTGF) levels in kidney [73]. These three proteins are shown to increase fibrosis in the diabetic kidney. Catalpol has significantly decreased Ang II and TGF-β1 concentration, which later preserves kidney function [73,77]. Hyperglycemia causes overexpression of TGF-β1 and stimulates the synthesis of ECM components in glomerulus through TGF-β1/smad pathway [78]. Activation of smad2/3 upregulates the expression for profibrotic genes such as collagen and CTGF. This activation leads to promoting fibrosis due to accumulation of ECM in the glomerulus and causes deterioration of glomerulus function [78]. Dong and Chen demonstrated that catalpol could reduce TGF-β1 concentration and down-regulate the mRNA of TGF-β1 expression in the diabetic kidney, which leads to decreased ECM components such as fibronectin, collagen IV, and CTGF [73]. Ang II has been shown to influence TGF-β1 to increase the synthesis of the ECM. Upon synthesis, TGF-β1 is first stored as a latent complex [79]. Ang II can stimulate the release of TGF-β1 from the latent form by upregulating thrombospondin-1. Otherwise, Ang II could stimulate the TGF-β1 promoter and increase the TGF-β1 gene transcription. Ang II also stimulates a chemokine, monocyte chemoattractant protein-1 (MCP-1), to enhance TGF-β1 transcription and synthesis. Ang II also directly upregulates TGF-β1 type 2 receptor [79]. The stimulatory effect of Ang II on TGF-β1 has been significantly reduced by catalpol and subsequently reduces the ECM accumulation, as shown in Figure 6 [73].

**Figure 7:**
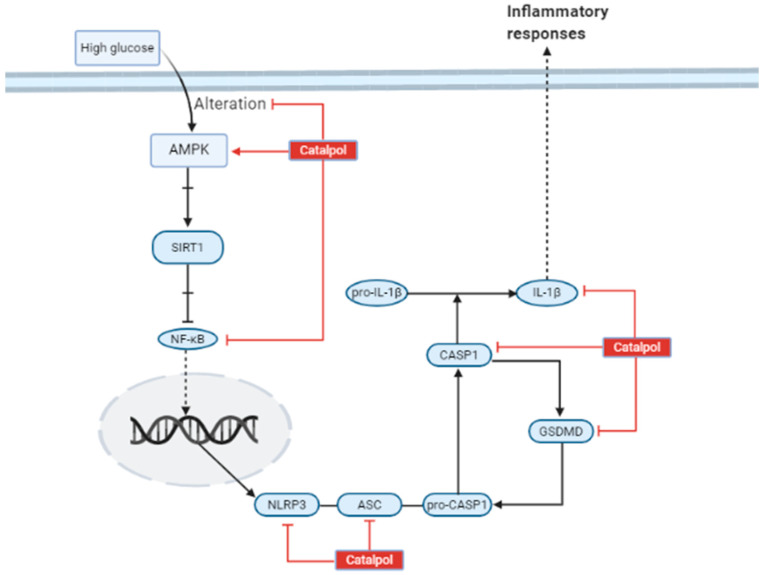
Catalpol on AMPK pathway in alleviating DN. Under normal circumstances, activation of AMPK could deactivate NF-κB. In DN, AMPK is being deactivated, which leads to activation of NF-κB. This could induce nuclear translocation and hence upregulate the pro-inflammatory cytokines such as NLRP3, ASC, and pro-CASP1. Later, caspase 1 is being cleaved and turns pro-IL-1β into IL-1β. IL-1β is secreted out of the membrane and causes inflammatory response. Catalpol acts to reverse the AMPK deactivation and hence downregulate NF-κB, leading to decreased inflammatory response.

Yang et al. showed that catalpol (10 mg/kg/day, i.p., 14 days) administration in male C57BL/6 mice down-regulates the growth factor receptor-bound protein 10 (Grb10) and activates insulin-like growth factor 1 receptor (IGF-1R) [72]. In DN, increased Grb10 expression is associated with deterioration of kidney function. Grb10 binds to IGF-1R and impairs IGF-1 signaling. IGF-1 has been shown to inhibit mesangial cell death, DNA damage due to hyperglycemia and promote repair of DNA [80]. Hence, in order to ameliorate kidney damage, catalpol has shown a decreased Grb10 concentration and increased IGF-1 signaling. In DN, the expression of caspase 3, an apoptotic protein, is upregulated, leading to increased cell death. Yang et al. have shown that catalpol is able to downregulate caspase 3 expression [72]. Moreover, catalpol has effectively preserved kidney function by augmenting AMP-activated protein kinase (AMPK), which is activated under depletion of energy [81]. Other than regulating energy levels, AMPK is linked with inhibition of nuclear factor-κB (NF-κB) inflammatory responses through downstream proteins produced by nicotinamide adenine dinucleotide (NAD+)-dependent protein deacetylase sirtuin 1 (SIRT1) and peroxisome proliferator-activated receptor-gamma coactivator 1 alpha (PGC-1α) [82]. SIRT1 and PGC-1α can interact with p65 subunit of NF-κB, which antagonizes the NF-κB inflammatory activity [82]. However, AMPK is altered in DN, leading to inflammatory responses. Chen et al. have studied the effect of catalpol on the AMPK signaling pathway in kidney and podocyte, which is illustrated in Figure 7 [76]. Catalpol (200 mg/kg, p.o.) has significantly upregulated AMPK and SIRT1 expression, whereas NF-κB is markedly downregulated. Further, catalpol has downregulated the pro-inflammatory mediators like IL-1β, caspase 1, Nod-like receptor protein 3 (NLRP3), Gasdermin D (GSDMD), and apoptosis-associated speck-like protein containing a C-terminal caspase-recruitment domain (ASC) [76].

Furthermore, catalpol is able to ameliorate podocyte injury and improve kidney function. In DN, hyperglycemia induces the apoptosis of podocytes, which are not replaced readily. As a result, the remaining podocytes change their size and shape in order to compensate for the lost podocytes, resulting in podocyte hypertrophy [83]. Urinary podocalyxin is increased in DN, and it acts as a specific marker for podocyte injury [75]. Chen et al. have shown that catalpol (60, 120 mg/kg) has significantly decreased the excretion of urinary podocalyxin in diabetic male C57BL/6J mice. Nephrin is a protein primarily found in the slit diaphragm between podocytes, and it plays a crucial role in maintaining the integrity of filtration membrane [83]. In DN, the expression of nephrin is downregulated, which is often associated with a disruption in filtration membrane and proteinuria [75]. Catalpol has significantly reversed the down-regulation of nephrin in kidneys [74,75]. Additionally, Chen et al. demonstrated that catalpol (100 mg/kg, p.o.) upregulated the expression of Wilms’ tumor 1 gene (WT1), which is the transcriptional factor of nephrin in male KK-Ay mice [75]. The excessive activation of RhoA, Cdc42, and Rac1 in podocytes promotes podocyte foot process effacement, albuminuria, and reorganization of podocyte skeleton [83]. Chen et al. have shown that catalpol could modulate the levels of RhoA and Cdc42 and thus stabilize the cytoskeleton [75]. Synaptopodin is an actin-associated protein that plays an important role in maintaining podocyte integrity. Synaptopodin expression is down-regulated in DN, leading to alteration of filtration membrane and proteinuria [83]. Chen et al. showed that catalpol is able to upregulate synaptopodin and preserve podocyte function. It has been shown that a lack of autophagy in podocytes is associated with proteinuria. Catalpol has also demonstrated an improved podocyte autophagy in diabetic kidney. Catalpol has improved autophagy through deactivating the mammalian target of rapamycin (mTOR) and activates transcription factor EB (TFEB) [75]. TFEB translocate into the nucleus and upregulate the transcription of autophagy genes. Chen et al. has demonstrated that catalpol can protect podocytes from inflammation and apoptosis by suppressing AGEs-RAGE pathway activation [74]. The binding of AGEs with RAGE has been proven to trigger several pathways such as MAPK, NADPH oxidase, and NF-κB. The activated pathways could induce pro-inflammatory responses and cell apoptosis [84]. Triggered by oxidative stress and environmental stress, c-Jun NH2-terminal kinase (JNK) facilitates apoptosis with upregulation of Bax expression and downregulation of Bcl-2. Bax acts as a pro-apoptotic protein, while Bcl-2 acts as an anti-apoptotic protein. Catalpol has decreased the expression of Bax and increased the expression of Bcl-2. [85].

### 3.2. Effects in Diabetic Encephalopathy

It has been proven that insulin is a vital hormone that consolidates memories in the frontal cortex and hippocampus. Insulin resistance or lack of insulin secretion can affect neuron survival, which later contributes to neurodegenerative diseases [86]. Otherwise, hyperglycemia can lead to neurotoxicity, causing damage to the brain [87]. Catalpol has demonstrated neuroprotective effect in diabetic encephalopathy (DE).

DE is manifested as impairment in learning and memory function. DE could lead to macroscopic changes in the hippocampus, causing CA1 pyramidal neurons to shrink or disappear [88]. The hippocampus plays an important role in consolidating memories. Thus, it is important to preserve the pyramidal neurons to improve cognitive function. Catalpol (5 mg/kg, i.p.) has significantly rescued the CA1 pyramidal neurons in STZ induced diabetic SD rats [89]. Wang et al. have also proven that catalpol (50 and 100 mg/kg, p.o.) could preserve CA1 pyramidal neurons significantly in STZ induced diabetic SD rats [16]. Further, Catalpol has significantly improved cognitive ability as compared to diabetic control by Morris water maze test and Y-type maze [16,89]. Under hyperglycemic condition, oxidative stress is increased together with an increment of reactive oxygen species (ROS). The level of antioxidant enzymes such as glutathione peroxidase (GSH-Px), superoxide dismutase (SOD), and catalase (CAT) is reduced. ROS can damage the neurons, causing diabetic encephalopathy [16,89]. Catalpol (5 mg/kg, i.p.; 50 mg/kg and 100 mg/kg, p.o.) can protect neurons by decreasing malondialdehyde (MDA) level and increasing the levels of GSH-Px, SOD, and CAT in experimental condition [16,89]. Zhou et al. demonstrated that catalpol (5 mg/kg, i.p.) has increased the level of protein kinase C gamma (PKCγ) and caveolin 1 (Cav-1) [89]. PKCγ is an isoform of protein kinase C (PKC) present in neurons and able to ameliorate oxidative stress and increase memory. Cav-1 is a membrane/lipid raft (MLR) that modulates synaptic plasticity. Synaptic plasticity is essential for sensing and transmitting signals between synapses. Decreased Cav-1 is related to the decreased synaptic plasticity and cognitive ability [90]. Protein and mRNA expression of both PKCγ and Cav-1 are downregulated in diabetic conditions, whereas catalpol can reverse the downregulation [89].

### 3.3. Effects in Diabetic Cardiovascular Conditions

Several studies have revealed that the prevalence of DM patients with any cardiovascular disease is at 32% [1]. Diabetic cardiomyopathy (DCM) is one of the significant complications of DM that causes hospitalization and death. Although DCM is asymptomatic at the initial stage, it can later develop into heart failure, coronary artery diseases, and myocardial infarction [91].

#### 3.3.1. Diabetic Cardiomyopathy

Under hyperglycemia, there is an increase in AGE formation, leading to activation of the AGE–RAGE pathway [92]. This pathway further activates NF-κB signaling, triggering intramyocardial inflammation by upregulating the expression of cell adhesion molecules such as intercellular adhesion molecule 1 (ICAM-1) and vascular cell adhesion molecule 1 (VCAM-1) and releasing inflammatory cytokines such as IL-1, IL-6, IL-8, and TNF-α [92]. Otherwise, the AGE–RAGE pathway upregulates the expression of TGF-β, which further activates the TGF-β/smad pathway. As a result, fibrosis occurs with an increase in expression of CTGF, fibronectin, and collagen. Hyperglycemia also induces activation of NADPH oxidase, which results in the formation of ROS [92]. The increased oxidative stress is associated with apoptosis and mitochondrial dysfunction. Apoptosis occurs with caspase activation, upregulation of Bax, and downregulation of Bcl-2. The increase in mitochondrial oxidative stress can cause abnormalities in mitochondrial ultrastructure and its function [93]. Increased oxidative stress also induces the activation of RAAS [94]. Once activated, Ang II is released and causes cardiac remodeling with increased fibrosis and cardiac hypertrophy [93]. In DM, there are increased serum free fatty acid and triglycerol levels, which leads to increased fatty acid utilization and oxidation. This is known to increase myocardial oxygen consumption and decreased cardiac efficiency. Increased fatty acid oxidation causes mitochondrial uncoupling and decreased ATP synthesis, leading to depletion in energy [93]. In DCM, there is an imbalance between uptake and storage of fatty acid that later causes lipotoxicity, leading to cause increased apoptosis, ROS formation, and cardiac remodeling [93]. Under insulin resistance, the insulin signaling in myocardial tissue is altered with impaired Akt and FOXO1 function [93]. Akt plays a significant role in inhibiting apoptosis, while FOXO1 is responsible for resistance in oxidative stress and DNA repair [93]. DCM is due to the myocardial cell death that leads to decrease in cardiac contractility and loss of cardiac function. DCM can cause myocardial infarction, atherosclerosis, or heart failure [95]. Overall, the molecular mechanism behind DCM is due to activation of the AGE–RAGE pathway, RAAS, and impaired insulin signaling pathway.

**Figure 8:**
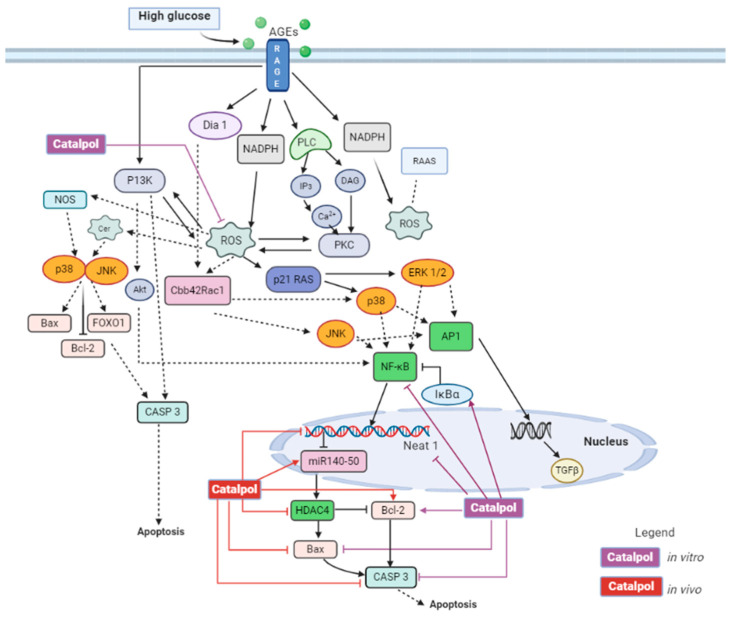
Cardioprotective mechanism of catalpol in diabetic cardiomyopathy. Under hyperglycemia, the production of AGE is increased, leading to activation of AGE–RAGE signaling pathway with increased ROS production and activation of NF-κB. IκBα acts as an inhibitor of NF-κB, which is able to inhibit apoptosis. Nuclear translocation of NF-κB binds to Neat 1 promoter. Neat 1 then inhibits expression of miR140-50; this then stimulates expression of HDAC4. As a result, apoptosis occurs with downregulation of Bcl-2 and upregulation of Bax and Casp-3. Catalpol has been shown to inhibit apoptosis in in vivo studies, where expression of Neat 1 is inhibited, leading to increased expression of HDAC4. Hence, there is increased upregulation of Bcl-2 and downregulation of Bax and Casp-3. In in vitro studies, catalpol has shown decreased production of ROS, which then decreases the activation of NF-κB. Catalpol also increased the expression of IκBα, which inhibits the activation of NF-κB. Catalpol inhibits Neat 1 expression, which causes upregulation of Bcl-2 and downregulation of Bax and Casp-3, leading to attenuation of apoptosis.

Zou et al. have demonstrated that catalpol (4 mg/mL) successfully inhibited cardiomyocyte death obtained from C57BL/6J mice [96]. In another study, catalpol (10 mg/kg) suppressed the cell apoptosis in adult male C57BL/6 mice, which was observed as reduced number of TUNEL-positive cells [97]. The anti-apoptotic effect of catalpol is due to the downregulation of caspase 3, Bax, and upregulation of Bcl-2 in cardiac tissue [96,97]. Molecular pathway of catalpol in in vitro and in vivo study was illustrated in Figure 8, where catalpol has inhibited apoptosis through ROS/NF-κB/Neat1 pathway and Neat1/miR-140e5p/histone deacetylase 4 (HDAC4) pathway. Catalpol has been shown to preserve cardiac function and alleviate morphological changes in myocardial cells. Zou et al. have reported that catalpol has significantly reduced the left ventricular end-diastolic pressure (LVEDP) and increased the left ventricular systolic pressure (LVSP), maximum rate of increased left ventricular pressure (+dp/dtmax), and maximal rate of decrease pressure during left ventricular relaxation (-dp/dtmax) significantly compared to DCM control group [97]. Histopathological examination of the heart tissue suggested that catalpol treatment has significantly decreased the myocardial cell degeneration, oedema formation, and attenuated the inflammatory cells infiltration into the myocytes. However, alteration of inflammatory markers such as TNF-α and IL-1β were not reported in this study.

#### 3.3.2. Myocardial Infarction

DM patients are estimated to have a 10% higher risk to experience an acute myocardial infarction (MI) [1]. DM patients that experience MI also have a significantly higher risk of mortality [98]. Hyperinsulinemia is associated with atherosclerosis development and progression [99]. In atherosclerosis, disrupted blood flow with decreased oxygen supply to cardiac cells results due to narrowing of coronary arteries. With increased plaque size, the blood flow could be fully blocked or there may be plaque rupture, which can form thrombosis and block the blood flow in smaller coronary capillaries leading to ischemia and MI [100,101]. One of the mechanisms of catalpol’s cardioprotective effects in MI is through ameliorating cell apoptosis caused by MI and ischaemia. Pre-treatment of catalpol (5 and 10 mg/kg, i.p.) in adult male Wistar rats prior to induction of MI has significantly reduced the percentage of apoptotic cells observed in TUNEL stained cells [102]. Similar findings were also reported by Huang et al., where catalpol (5 mg/kg, i.p.) pre-treatment in adult male SD rats has reduced apoptosis [103]. In glucose-deprived H9c2 cardiomyocytes, catalpol (0.1, 1, 10 μg/mL) treatment for 24 h has significantly inhibited apoptosis such as cell shrinkage, DNA condensation, fragmentation of nucleus, and decreased mitochondrial membrane potential [104]. Catalpol pretreatment reversed the downregulation of Bcl-2 and upregulation of Bax, caspase 3, and caspase 9 in cardiac cells, which could be linked to its anti-apoptosis properties [102,104]. Cardioprotective role of catalpol is also linked to its antioxidant properties, where catalpol could suppress oxidative stress induced by MI. During ischemic/reperfusion, ROS production overwhelms the antioxidant enzyme levels in myocardial tissue. This causes increased oxidative stress in cardiac cells, which later leads to increased inflammation or cell death [105]. Several reports suggested that with catalpol pre-treatment, MDA level was significantly decreased together with a marked elevation of SOD level [103,104,106,107]. Under acute reperfusion, superoxide levels are increased together with overexpression of gp91phox. Superoxide is generated from NADPH oxidase (NOX), which is made up mainly by gp91phox. Catalpol has shown to reverse the increment of superoxide levels and overexpression gp91phox in cardiac tissue with ischemia/reperfusion insult [103]. The role of catalpol in promoting autophagy and mitophagy after MI was reported in experimental models. Autophagy acts to protect cells from apoptosis when there is increased oxidative stress [108]. Mitophagy is a specific autophagy process that occurs in damaged mitochondrial [109]. In an in-vitro study, Lin et al. have shown that catalpol (1 and 10 μg/mL) has increased autophagy and mitophagy in cardiomyocytes [104]. Catalpol has increased the concentration of lysosome and LC-3. Monodansylcadaverine (MDC) is a specific marker to autophagolysosome and is increased upon catalpol treatment. Catalpol also has increased expression of autophagy specific proteins such as Beclin 1, LC3 I, LC3 II, Parkin, autophagy-related 5 (Atg5), and decreased expression of PTEN-induced kinase (PINK) and P61. The autophagy and mitophagy promoting effect of catalpol is possibly linked to estrogen receptor binding ability, which was completely blocked in the presence of tamoxifen (TAM), a selective estrogen modulator. TAM exhibited blockage of catalpol function in inhibiting apoptosis, decreasing Bax and caspase 3, increasing Bcl-2 and effects of catalpol on autophagy specific proteins such as Beclin 1, LC3 I, LC3 II, Parkin, Atg5, PINK, and P61 [104].

**Figure 9:**
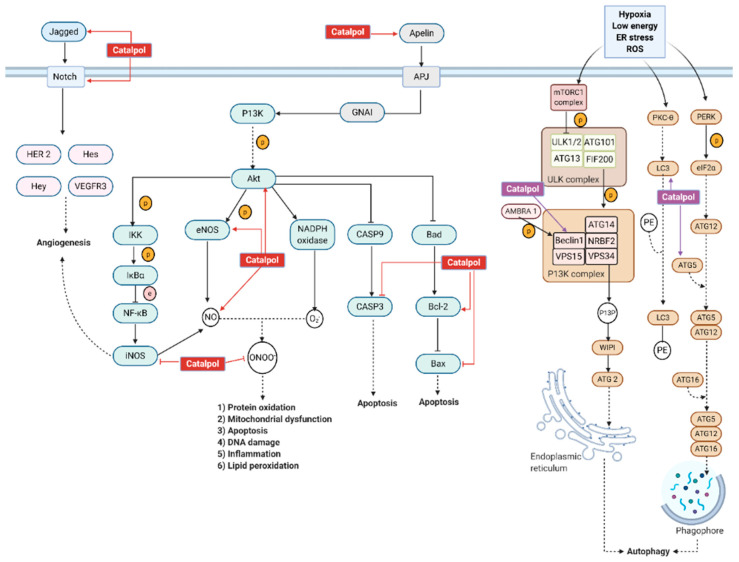
Cardioprotective mechanism of catalpol in MI. Catalpol is able to upregulate Notch/Jagged pathway to stimulate angiogenesis after MI. Catalpol could also upregulate Apelin/APJ signaling pathway to exert cardioprotective effect. Activation of APJ later activates P13K/Akt pathway. APJ is involved in regulating Akt/eNOS pathway that could stimulate cell proliferation, angiogenesis, and DNA repair. Moreover, activation of Akt could scavenge the superoxide anion (free radical). NO is an antioxidant and could also exert vasodilation effect. However, under increment of free radicals, NO could react with O2∙ to form ONOO∙. ONOO∙ could further contribute to increase oxidative stress. This later leads to protein oxidation, mitochondrial dysfunction, apoptosis, DNA damage, inflammation, and lipid peroxidation. Catalpol is able to decrease oxidative stress by reducing O2∙, increasing NO, and reducing formation of ONOO∙. Akt activation could prevent apoptosis by inhibiting caspase 9 and Bad. Catalpol further exerts its cardioprotective effect by inhibiting caspase 3 and Bax and upregulating Bcl2. Catalpol is able to upregulate autophagy proteins such as Beclin1, AGT5, and LC3 that promotes autophagy.

Catalpol (10 mg/kg, i.p.) has reversed the cardiac injury in isoproterenol (ISO)-induced myocardial damage in Wistar rats. ISO control rats have edema, necrosis, and inflammatory cells infiltration. However, rats pretreated with catalpol have significant reduction of cardiac injuries with smaller or mild inflammatory cells infiltration. Catalpol has shown to downregulate gene and protein expression of inflammatory mediators such as TNF-α and IL-1β [106]. Zeng et al. used 2,3,5-Triphenyltetrazolium chloride (TTC) staining to study the area of infarction in cardiac cells. In catalpol (10, 20, and 40 mg/kg, p.o.) treated rats, the cardiac cells have smaller white areas in dose dependent manner, as observed microscopically using TTC staining. This indicates catalpol treatment has reduced the infarct area in MI [107]. Huang et al. have also proven that catalpol treatment could significantly reduce infarct size in myocardial cells. Microscopic examination of cardiac cells using H&E staining showed a disrupted arrangement, swollen muscle fiber, shrunk nucleus, and infiltration of inflammatory cells in MI cardiac tissue. Infarcted cardiac muscle cells show necrosis with disappeared and dissolved nucleus [103]. Catalpol (10 and 20 mg/kg, i.p.) have partially inhibited the infiltration of inflammatory cells and myocardial muscle fibers damage. At higher dose of catalpol (40 mg/kg, i.p.), the cardiac cells were mostly intact and myocardial muscle fibers showed more regular arrangement and decreased inflammatory cells infiltration [106]. Catalpol has not only improved/protected the molecular and microscopic character of cardiac tissues, but it has also demonstrated an enhanced physiological function of the heart by preserving the left ventricular function. Following a MI, there is an increase in LVEDP, decrease in LVSP, + dp/dtmax and - dp/dtmax. Bi et al. have demonstrated that catalpol (5 mg/kg, i.p.) pretreatment could prevent decrease in LVEDP and increase in + dp/dtmax. Catalpol (10 mg/kg, i.p.) pretreatment has prevented an increase in LVEDP and a decrease in LVSP, + dp/dtmax and - dp/dtmax caused by MI. In addition, Catalpol (10 mg/kg, i.p.) reverses the decline of systolic, diastolic, and mean blood pressure. Bi et al. have concluded that the cardioprotective effect is due to modulation of the Apelin/APJ signaling pathway as shown in Figure 9 [106]. Following MI with reperfusion, Huang et al. found out that catalpol increased LVSP, + dp/dtmax, and - dp/dtmax. However, effect on LVEDP is not reported in the study. Huang et al. have proposed that catalpol acts on P13K/Akt/eNos pathway to exert a cardioprotective effect as shown in Figure 9 [103]. Zeng et al. found out catalpol has reversed the abnormal elevation of the S-T segment in a dose-dependent manner. Further, catalpol provides a cardioprotective effect through improving angiogenesis via endothelial progenitor cells (EPC) and Notch1/Jagged1 pathway, as shown in Figure 9 [107].

In MI, a few cardiac specific biomarkers including Troponin T, Troponin I, creatine kinase (CK), and lactate dehydrogenase (LDH) are elevated in blood upon cardiac injury [110]. Several reports have shown that catalpol pretreatment significantly reduced the levels of CK and LDH in MI models, which suggests its protection against the myocardial injury caused by ischaemia [103,104,106,107]. Catalpol could also promote the survival of bone mesenchymal stem cells (BMSC) after transplantation into myocardial tissue after MI. Cardiac damage due to MI has been known to cause irreversible damage and loss of myocardial cells. However, the interventions available currently could not rescue the damaged cells after MI except BMSC transplantation. BMSC is mesenchymal stem cells (MSC) that could be easily cultivated and easily separated, are highly stable for amplification, have low immunogenicity, and are easily obtained. MSC exhibited self-renewal properties and is able to differentiate into bone, cells, cartilage, muscle, bone marrow, or fat upon stimulation. BMSC is transplanted into myocardial tissue, which could differentiate into cardiomyocytes [111]. However, the survival of BMSC is low. This is because there is a lack of blood supply and poor nourishment, which fails to demonstrate the cardioprotective effect [112]. Ju et al. have shown that catalpol has improved the BMSC survival and cardiac function in MI Wistar rats where BMSC were pre-treated with catalpol (49 μg/mL, 24 h) prior to transplantation. The survival of BMSC in cardiac tissues is enhanced by catalpol as observed with PKH26 dye. Catalpol pre-treatment has also enhanced the effect of BMSC to reverse the fibrosis and morphological changes in myocardial cells, as shown in HE and Masson stain. BMSC has alleviated cell apoptosis, and catalpol pre-treatment further enhanced the anti-apoptotic effect, as observed with TUNEL and myosin staining. BMSC has elevated left ventricular ejection fraction (LVEF) and left ventricular fractional shortening (LVFS), which was enhanced with catalpol pretreatment. MI caused an increased left ventricular end-systolic dimension (LVESD) and left ventricular end-diastolic dimension (LVEDD), which was reversed by BMSC, and this effect of BMSC was further enhanced by catalpol pretreatment [113]. BMSC plays an important role in order to improve overall cardiac function by releasing VEGF, a pro-angiogenesis factor to the surrounding area to stimulate angiogenesis [112]. Angiogenesis is a crucial factor to cut down the ischemic area and increase perfusion of blood into cardiac cells after MI [114]. Ju et al. have shown that BMSC could increase both the protein levels of VEGF-A and CD31, which are the specific markers of endothelial cells. The effect was further enhanced by catalpol pre-treatment [113].

#### 3.3.3. Diabetic Atherosclerosis and Arrhythmias

DM is a risk factor contributing to the development of atherosclerosis [115]. Hyperglycemia and hyperinsulinemia are known to promote atherosclerosis [99]. The uptake of glucose by endothelial cells is independent of glucose transporter-4 (GLUT-4). Hence, in hyperglycemic conditions, an excess amount of glucose can enter endothelial cells [116], resulting in a large amount of ROS generation and AGE production, further stimulating the AGE–RAGE signaling pathway [117]. Through this pathway, inflammatory cytokines, growth factors, and collagen are produced that lead to the recruitment of inflammatory cells, platelet aggregation, angiogenesis, fibrosis, and increased vascular permeability [118]. Not many studies have described the protective effect of catalpol on diabetic atherosclerotic animal model. Liu et al. have induced diabetic atherosclerosis in male New Zealand White diabetic rabbits by using alloxan (100 mg/kg, i.v.) together with hyperlipidemic diet [119]. In this study, Liu et al. showed that catalpol (50 mg/kg/d, p.o.) has reduced the body weight, blood glucose, and HOMA-IR and increased the plasma insulin level of diabetic rabbits. Further, catalpol reversed the increased MDA concentration, protein carbonyl groups (PCG), and AGE levels found in diabetic rabbits. In addition, catalpol has increased the antioxidant enzymes (such as SOD and GSH-Px) and reduced the expressions of inflammatory cytokines significantly [119]. Aggregation of ECM and fibrosis is commonly seen in diabetic atherosclerotic lesion. Upregulation of TGF-β1 modulates the fibrosis pathway and induces the production of ECM such as Collagen IV (Col-IV) [120]. In this study, Liu et al. showed that catalpol has significantly decreased both the mRNA and protein levels of TGF-β1 and Col-IV [119]. In diabetic atherosclerosis, the plaque has increased thickness of intima and media and increased infiltration of VSMC and macrophages. However, catalpol has reversed all these effects significantly [119]. These results suggest that catalpol is able to ameliorate diabetic atherosclerosis by decreasing oxidative stress, infiltration of inflammatory cytokines, and fibrosis, resulting in a delay in formation of atherosclerosis plaque.

DM patients with poorly controlled blood glucose level have higher rates of having arrhythmia manifested with irregular heartbeat [121]. Catalpol has demonstrated a cardioprotective effect against arrhythmia. Human induced pluripotent stem cells (iPSC) are cultured and differentiated into cardiomyocytes. Then, catalpol is incubated with iPSC for 24 h. Catalpol (10 and 100 μM) significantly improved the cell viability and decreased the LDH levels in arrhythmia [122]. Catalpol (10 and 100 μM) significantly reduced lipid peroxidation in iPSC and also successfully attenuated oxidative stress by increasing SOD, Gpx, and GSH. The activities of caspase 3 and caspase 9 are also decreased by catalpol, indicating catalpol has attenuated apoptosis [122]. However, these activities need to be further established using in vivo arrhythmia model.

### 3.4. Effects in Other Diabetes Related Complications

Individuals with DM are associated with an increased risk of diabetic osteoporosis (DOP) [123]. Insulin plays an important role in stimulating the proliferation and differentiation of osteoblasts. There is lack of insulin production in T1DM, and hyperglycemia leads to an increased incidence of bone fractures [123,124]. Hence, both T1DM and T2DM individuals are known to have a higher risk to be diagnosed with DOP. Catalpol exerted protective effects on DOP by stimulating the osteoblast proliferation and differentiation to increase bone formation in an in vitro study [125]. Cheng et al. have incubated osteoblast MC3T3-E1 cells in high glucose to imitate DOP. Catalpol (1, 2, and 4 mg/mL) has increased the proliferation and differentiation of MC3T3-E1 cells in a dose-dependent manner through the Wnt/β-catenin signaling pathway. Under high glucose, this pathway is disrupted, which causes excessive bone resorption. Activation of Wnt/β-catenin signaling pathway upregulates the expression of runt-related transcription factor 2 (RUNX2), Collagen I, osteocalcin (OCN), bone morphogenetic protein 4 (BMP4), and bone morphogenetic protein 7 (BMP7) [125]. RUNX2 is a transcription factor required for osteoblast development. Collagen I is the main collagenous compound in bone, and it is essential to evoke differentiation of mesenchymal stem cells (MSC) into osteoblasts. OCN is a protein secreted by osteoblasts and known to regulate insulin production. BMP is a precursor that stimulates the differentiation of MSC into osteoblasts [126,127]. Thus, catalpol has shown an improved proliferation and differentiation in osteoblasts, although this needs to be further established using in vivo models.

Diabetic retinopathy (DR) is one of the complications caused by DM. Without early diagnosis and treatment, DR ultimately causes sight impairment and blindness [1]. In the early stage of DR, blood vessels in the retina are dilated due to hyperglycemia, and this increases the blood flow to the retina. This microvascular dysfunction leads to increased retinal metabolism and death of pericytes inducing microaneurysm. Pericytes participate in maintaining angiogenesis and regulating blood flow [128]. Excess glucose level increases sorbitol level through activation of polyol pathway, and an excess amount of sorbitol is accumulated among the cells, which causes osmotic stress and damage [129]. Hyperglycemia causes the accumulation of AGE and activates AGE–RAGE pathway. Through this pathway, ROS is generated that exerts oxidative stress and recruits inflammatory cytokines such as TNFα, IL-1, IL-6, and IL-8 with an accumulation of adhesion molecules such as VCAM-1 and ICAM-1 by activating NF-κB [129]. Retinal neurodegeneration took place in retinal ganglion cells (RGCs) responsible for forming vision in the retina. However, hyperglycemia is known to induce apoptosis of RGCs [130]. Catalpol has exerted protective effects in DR with in vitro study. Shao et al. have cultured retinal ganglion cells (RGCs) in high glucose concentration as a DR model [131]. It has been shown that lactate dehydrogenase (LDH), a marker for tissue damage, is increased in DR. Catalpol (2 mm) has shown a marked attenuation of LDH activity and increased the survival rate of RGCs. This protective effect of catalpol was linked to its ability to inhibit oxidative stress and NF-κB downregulation [131].

## 4. Conclusions

Antidiabetic/hypoglycaemic effect of catalpol was possibly linked to the promotion of insulin signaling in all the insulin-sensitive peripheral organs, improved mitochondrial function in skeletal muscle, suppressing oxidative stress, and inflammation. In skeletal muscles, catalpol increased the insulin sensitivity and glucose utilization through IRS-1/PI3K/AKT/GLUT4, AMPK/PGC-1α/TFAM, and AMPK/SIRT1/PGC-1α/PPAR-γ IRS-1 signaling and improved myogenesis, mitochondrial biogenesis, function, and respiration. The underlying mechanisms of catalpol in the liver are primarily linked to the promotion of glycogenesis and suppression of gluconeogenesis through IRS-1/PI3K/AKT pathway; phosphorylation of AMPK, GSK3β, and FOXO1; activation of β-endorphin; and translocation of GLUT2. In adipose tissue, catalpol attenuated the inflammation by suppressing M1-proinflammatory factor, NOX4 activity, p-JNK, p-IKκB, and NF-κB and increasing M2-anti-inflammatory factor. Reduced inflammation is linked to improved insulin sensitivity and glucose uptake in the adipose tissue. Studies suggested that catalpol increases the autocrine/paracrine function of the pancreas in releasing insulin via IRS-1/AKT/GLUT2 expression and improving antioxidant enzyme levels.

Catalpol has demonstrated significant protection/treatment against diabetic complications, primarily through its anti-oxidative, anti-apoptotic, anti-inflammatory, and anti-fibrotic properties and promoting autophagy. In DN, catalpol reduced thickening and matrix deposition in the glomerular basement membrane by preventing the glycoprotein deposition, podocyte injury, and cell death. Besides all primary activities mentioned above, catalpol decreased Ang II, TGF-β1, and CTGF levels; upregulated AMPK/SIRT1; and downregulated NF-κB expression in kidney. Catalpol’s significant neuroprotective action against diabetic encephalopathy is due to its modulation of synaptic plasticity and antioxidative property. Catalpol showed significant cardioprotective effects against diabetes cardiomyopathy, myocardial infarction, atherosclerosis, and arrhythmia. Catalpol showed the antiapoptotic activity mediated via. ROS/NF-κB/Neat1 and Neat1/miR-140e5p/histone deacetylase 4 (HDAC4) pathway, modulated the Apelin/APJ signaling pathway, and overall improved the cardiac performance. Catalpol exerted an anti-osteoporotic effect in diabetic osteoporosis by stimulating osteoblast proliferation. The protective effect against diabetic retinopathy is also shown by catalpol in preventing neurodegeneration of retinal ganglion cells. Currently, it can be concluded that catalpol has marked efficacy against diabetes and its complications, but the safety and toxicity studied of catalpol need to be investigated before it can be considered as a potential molecule for human studies.

## Data Availability

The data presented in this study are openly available.
